# The value of whole-lesion histogram analysis based on field‑of‑view optimized and constrained undistorted single shot (FOCUS) DWI for predicting axillary lymph node status in early-stage breast cancer

**DOI:** 10.1186/s12880-022-00891-6

**Published:** 2022-09-10

**Authors:** Shu Fang, Jun Zhu, Yafeng Wang, Jie Zhou, Guiqian Wang, Weiwei Xu, Wei Zhang

**Affiliations:** 1 Department of Radiology, Changzhou Hospital of Traditional Chinese Medicine, Changzhou City, 213000 Jiangsu Province China; 2 Department of Pathology, Changzhou Hospital of Traditional Chinese Medicine, Changzhou City, 213000 Jiangsu Province China

**Keywords:** Breast cancer, Axillary lymph node, Histogram, FOCUS DWI

## Abstract

**Background:**

This study aims to estimate the amount of axillary lymph node (ALN) involvement in early-stage breast cancer utilizing a field of view (FOV) optimized and constrained undistorted single-shot (FOCUS) diffusion-weighted imaging (DWI) approach, as well as a whole-lesion histogram analysis.

**Methods:**

This retrospective analysis involved 81 individuals with invasive breast cancer. The patients were divided into three groups: N_0_ (negative ALN metastasis), N_1–2_ (low metastatic burden with 1–2 ALNs), and N_≥3_ (heavy metastatic burden with ≥ 3 ALNs) based on their sentinel lymph node biopsy (SLNB) or axillary lymph node dissection (ALND). Histogram parameters of apparent diffusion coefficient (ADC) depending basically on FOCUS DWI were performed using 3D-Slicer software for whole lesions. The typical histogram characteristics for N_0_, N_1–2_, and N_≥ 3_ were compared to identify the significantly different parameters. To determine the diagnostic efficacy of significantly different factors, the area under their receiver operating characteristic (ROC) curves was examined.

**Results:**

There were significant differences in the energy, maximum, 90 percentile, range, and lesion size among N_0_, N_1–2_, and N_≥ 3_ groups (*P* < 0.05). The energy differed significantly between N_0_ and N_1–2_ groups (*P* < 0.05), and some certain ADC histogram parameters and lesion sizes differed significantly between N_0_ and N_≥3_, or N_1–2_ and N_≥3_ groups. For ROC analysis, the energy yielded the best diagnostic performance in distinguishing N_0_ and N_1–2_ groups from N_≥3_ group with an AUC value of0.853. All parameters revealed excellent inter-observer agreement with inter-reader consistencies data ranging from0.919 to 0.982.

**Conclusion:**

By employing FOCUS DWI method, the analysis of whole-lesion ADC histogram quantitatively provides a non-invasive way to evaluate the degree of ALN metastatic spread in early-stage breast cancer.

## Background

Breast cancer is the most prevalent cancer type in females globally and seriously endangering women's health [1]. Accurate identification of the extent of axillary lymph node (ALN) involvement plays a crucial role for patients with breast cancer because it is an essential prognostic factor and influences the clinical therapeutic schedule [2]. According to ACOSOG Z0011 trial, the 10-year overall survival rate of patients with early-stage invasive breast cancer who had 1 or 2 sentinel lymph nodes metastases was not lower if only sentinel lymph nodes dissection (SLND) but not axillary lymph node dissection (ALND) was performed [3]. Compared with ALND, it was reported that SLND had fewer complications and might offer precise staging data without raising the risk of localized recurrence or lowering survival [4]. However, SLND is also an intrusive technique and has some complications, including shoulder dysfunction, upper arm numbness, nerve damage, and lymphedema [5]. In fact, most breast cancer patients had negative ALN metastasis in early-stage [6], and 43–65% of patients with positive SLN suffered from overtreatment of ALND because there was no additional non-sentinel lymph node metastasis [7]. Therefore, it is required to forecast the magnitude of ALN involvement with early-stage breast cancer patients in a non-invasive way.

As functional imaging, diffusion-weighted imaging (DWI) does not require intravenous contrast agent injections containing gadolinium and is widely used to assess breast tumors [8]. Quantitative parameter of apparent diffusion coefficient (ADC) values generated depending on DWI were associated with microenvironmental and microstructural changes in cancers [9]. Previous studies have reported that quantitative ADC values were correlated with breast cancer predictive factors, comprising molecular subtypes, histological grade, and recurrence risk [10, 11]. It has been demonstrated that early-stage invasive breast cancer with ALN metastasis had lower tumor ADC values [12]. Choi et al*.* found that peritumoral maximal ADC/tumoral ADC was independently associated with sentinel lymph node (SLN) metastasis [13]. However, most previous studies obtaining ADC values only mapped the regions of interest (ROI) on single or multiple levels of the lesion, and it may be unable to adequately portray the variety of complete tumors. As an emergent image processing technique, histogram analysis based on the probability distribution of gray pixel value can provide more quantitative information on tumor heterogeneity assessment parameters [14]. Lately, whole-lesion ADC histogram analysis has been used and has proven beneficial in assessing aggressiveness and eventual prognosis of breast cancer [15, 16].

Moreover, it is noteworthy that higher-resolution images are essential for whole-lesion histogram analysis [17]. The field-of-view (FOV) optimized and constrained undistorted single-shot (FOCUS) has a higher spatial resolution and clearer contrast resolution compared with spin-echo single excitation (SS-EPI) sequence, which uses a 2-dimensional spatially selective echo-planar radiofrequency excitation pulse technique [18]. FOCUS technique has been applied to the spinal tumor [19], pancreatic tumors [20], and cervical carcinoma [21]. These studies believed that FOCUS DWI offers more accurate ADC values, possibly due to their less partial volume effect and clearer anatomic details. To date, as far as we know, the whole-lesion histogram parameters of the main tumor as determined by FOCUS DWI have not been properly described for evaluating ALN involvement extent in breast cancer. Hence, we aimed at evaluating the utility of whole-lesion ADC histogram parameters depending on FOCUS DWI method in predicting the extent to which ALNs are involved in breast cancer in its early stages.

## Methods

### Patients

This retrospective study was approved by the Ethics Review Committee of Changzhou Traditional Chinese Medicine Hospital (2019-LL-03(JS)) and the need for signed informed consent was waived. All methods were carried out in accordance with relevant guidelines and regulations. From March 2019 to April 2022, this research enrolled 178 breast cancer patients. The following were the inclusion criteria: (1) patients with complete clinical data; (2) patients who underwent preoperative MR examinations, including FOCUS DWI imaging; (3) patients who had breast surgery along with sentinel lymph node biopsy or ALN dissection, and only stage I or II invasive breast cancers were involved. The exclusion criteria included the following: (1) non-mass-like enhancement on dynamic contrast-enhanced (DCE) MRI; (2) poor image quality with obvious motion artifacts; (3) patients with lesion size smaller than 10 mm; (4) a history of radiotherapy, chemotherapy, endocrine therapy and surgery before MRI examination. Finally, 81 patients (range: 25–78 years; mean age 56.05 ± 11.28 years) were chosen for this research.

### MRI technique

All magnetic resonance imaging (MRI) examinations were done using a 3.0 T MRI system (SIGNA Pioneer; GE Healthcare) with a dedicated eight-channel bilateral breast coil. In the prone posture, the subject is put, and bilateral breasts are naturally draped.

***MRI unenhanced scanning.*** An axial T1-weighted imaging parameters included the following: TR/TE, 696 ms/shortest; section thickness, 4 mm; gap, 1 mm; FOV, 360 × 360 mm; matrix, 320 × 256; number of signals acquired [NEX]. Fat suppression T2WI imaging parameters included the following: TR/TE, 5537/85 ms; section thickness, 4 mm; gap, 1 mm; FOV, 360 × 360 mm; matrix, 384 × 256; bandwidth, 62.5HZ. FOCUS DWI scanning parameters included the following: TR/TE, 7890 ms/shortest; slice thickness, 4 mm; gap, 1 mm; FOV, 360 × 180 mm; matrix, 160 × 70; NEX, 6; b value, 0 and 800 s/mm^2^.

***Dynamic contrast-enhanced MRI.*** DCE MRI scanning parameters included the following: TR/TE, 4.5/2.1 ms; flip angle, 15°; FOV, 360 × 360 mm; matrix, 360 × 360; layer thickness, 1 mm. Intravenously, the contrast agent (gadolinium chelate) was administered at a rate of 2.0 mL/s at a dosage of 0.1 mmol/kg, followed by flushing with a 20 mL saline solution. A mask was scanned prior to injection. Following injection, six-time phases of volume imaging were collected continuously.

### Histogram analysis

The selected FOCUS DWI pictures were exported in DICOM format from PACS workstation for histogram analysis. Throughout the export process, all pictures were altered to maintain a uniform window width and level. All the MR images were retrospectively reviewed by two radiologists with five and ten years of expertise in breast tumor imaging diagnosis. The radiologists were blinded to the patients’ pathologic information except for the diagnosis of invasive breast cancer. In case of multicenter or multifocal tumors, the two radiologists would reach a final decision by consensus.

ROIs were independently delineated by the same two radiologists. ROIs were manually drawn around the whole tumor margin on each level of b = 800 s/mm^2^ FOCUS DWI map using open-source software (http://www.vusion.com.cn/) and then copied onto ADC maps, by the utility of DCE sequence as a reference standard. As a consequence, a three-dimensional volume of interest for the whole tumor is created. ADC histogram parameters for the entire volume were calculated using 3D-Slicer software, including any cystic or necrotic parts and hemorrhagic components (https://www.slicer.org/). Finally, the following parameters were derived: the minimum, median, mean, maximum, kurtosis, skewness, energy, 90 percentile, 10 percentile, and range.

### Histopathologic analysis

Histological grade, PR status, ER status, HER2 status, Ki-67, and axillary lymph node status were obtained from histopathological reports of surgical specimens recorded in the hospital's PACS medical record system. The proportion of ER and PR positive cells was more than 1% and recorded as positive. If IHC staining score was HER2 (3 +), or FISH gene showed amplification, it was recorded as HER‑2 positive. High expression of Ki-67 was defined as the percentage of positive tumor cell nuclear immunostaining in the background level under a high magnification microscope ≥ 14%, and < 14% was low expression.

### Statistical analysis

The measured data were analyzed using SPSS (version 22.0; IBM, Armonk, NY, USA). Kolmogorov–Smirnov test was used to determine normality. Mean and standard deviation were used to describe normally distributed data; the median was used to describe skewed data (interquartile range). Chi-square test, Kruskal–Wallis, and one-way analysis of variance (ANOVA) were employed to compare clinical characteristics among various ALN groups. The intraclass correlation coefficient (ICC) was used to determine the interobserver variability of ADC histogram parameters based on FOCUS DWI method. When histogram parameters were normally distributed, one-way ANOVA was used to compare the three groups, and when they were not normally distributed, Kruskal–Wallis H test was used. The least significant difference (LSD) test (homogeneity of variance) or Mann–Whitney U test (heterogeneity of variance) was employed for posthoc pairwise comparisons. According to diagnosis results, to determine the diagnostic performance of each significant parameter in forecasting ALN metastasis in breast cancer, receiver operating characteristic (ROC) curve studies were conducted. *P* < 0.05 was considered statistically significant.

## Results

### Clinicopathological features

**Fig. 1:**
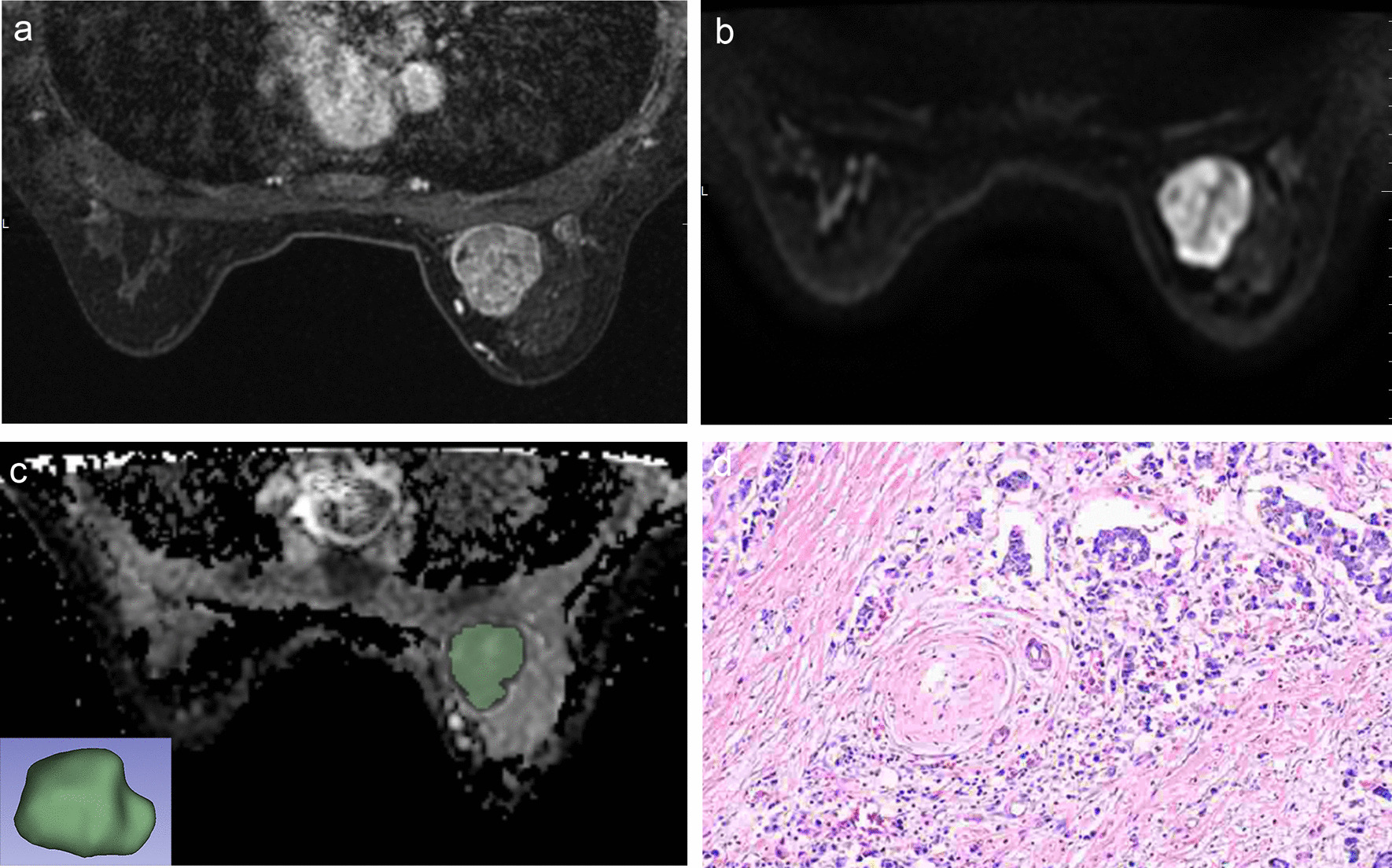
A 49-year-old woman with negative ALN metastasis. **a** Axial T1-weighted contrast-enhanced MR image shows an oval enhancing mass. **b** FOCUS DWI MR image (b value of 800 s/mm^2^) shows high signal intensity mass. **c** The corresponding ADC map copied ROI from FOCUS DWI image to obtain the ADC histogram, and a 3D-ROI covering the whole lesion. **d** Histopathological hematoxylin & eosin staining (H&E) (× 40) image shows right breast invasive carcinoma with the histologic score was 3. Immunohistochemical staining revealed positive expression of ER and PR and a high Ki-67 index (35 ~ 50%)

**Fig. 2:**
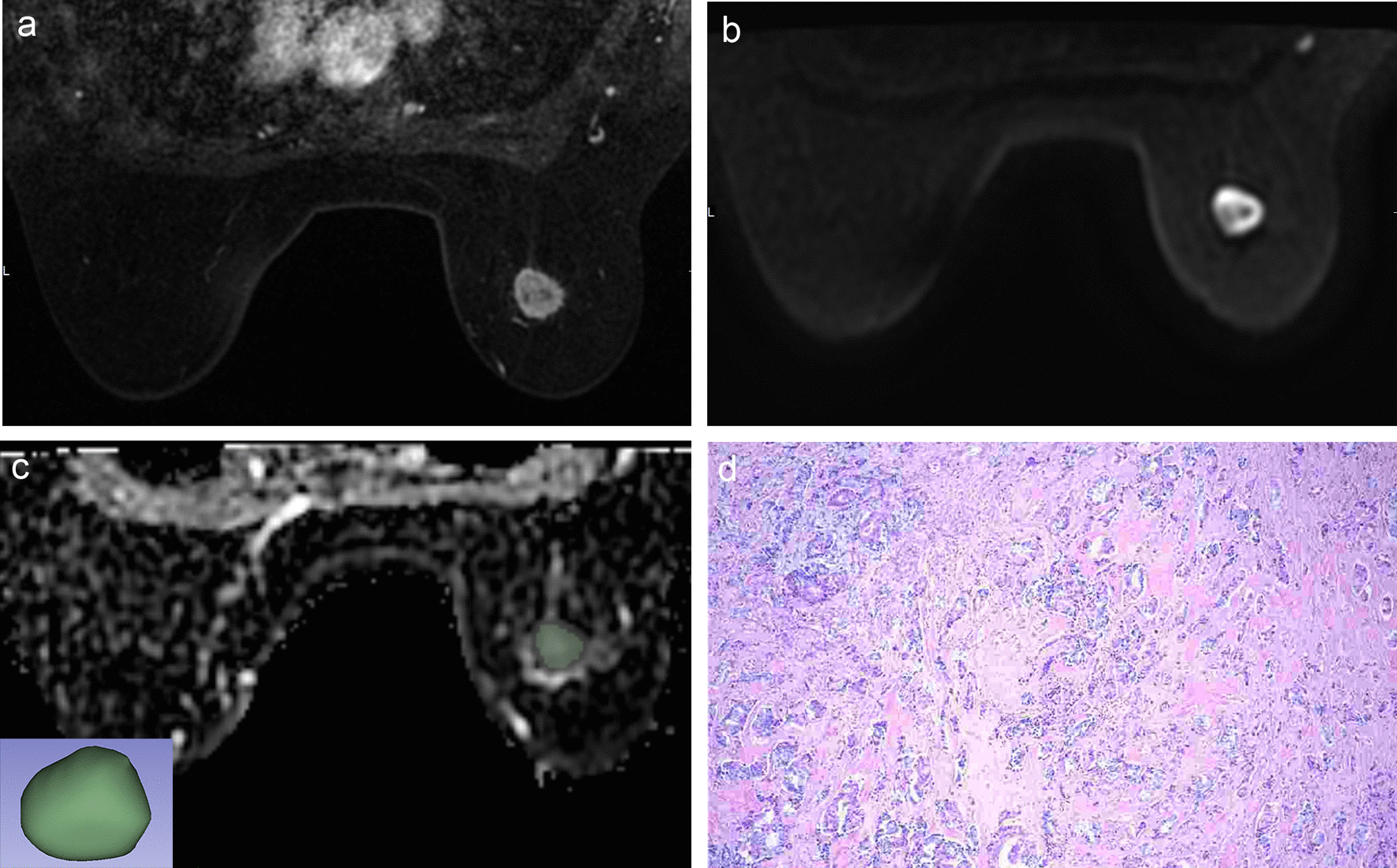
A 55-year-old woman with 1 axillary node metastasis was found in 5 resected nodes. **a** Axial T1-weighted contrast-enhanced MR image shows an irregular enhancing mass. **b** FOCUS DWI MR image (b value of 800 s/mm^2^) shows high signal intensity mass. **c** The corresponding ADC map copied ROI from FOCUS DWI image to obtain the ADC histogram, and a 3D-ROI covering the whole lesion. **d**) Histopathological H&E (× 100) image shows right breast invasive carcinoma with the histologic score was 2. Immunohistochemical staining revealed positive expression of ER and PR and a high Ki-67 index (20%)

**Fig. 3:**
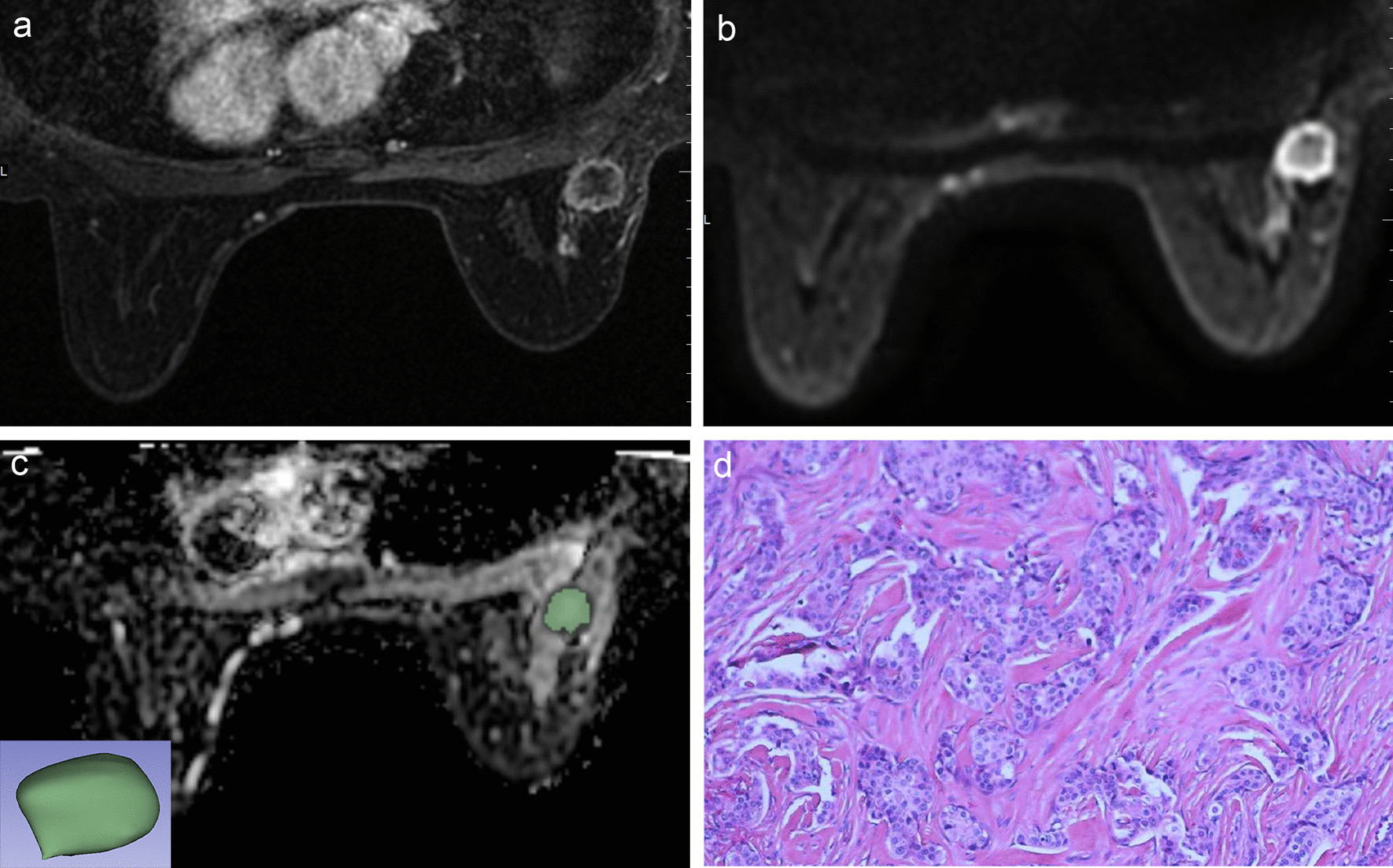
A 51-year-old woman with 5 axillary node metastasis was found in 25 resected nodes. **a** Axial T1-weighted contrast-enhanced MR image shows a round enhancing mass. **b** FOCUS DWI MR image (b value of 800 s/mm^2^) shows high signal intensity mass. **c** The corresponding ADC map copied ROI from FOCUS DWI image to obtain the ADC histogram, and a 3D-ROI covering the whole lesion. **d** Histopathological H&E (× 40) image shows right breast invasive carcinoma with the histologic score was 2. Immunohistochemical staining revealed positive expression of ER and PR and a low Ki-67 index (5%)

All 81 patients underwent SLND or ALND, and histopathological results revealed that 45patients had negative ALN (N_0_), 16 had 1–2 metastatic ALNs (N_1–2_), and 20 had three or more metastatic ALNs (N_≥3_). No significant differences were shown in age, tumor position, ER, PR, HER2, Ki-67, molecular subtype, and histologic grade among N_0_, N_1–2_, and N_≥ 3_ groups (*p* > 0.05). The lesion size significantly differed among the three groups (*p* = 0.000). The patient and tumor features are summarized in Table 1 and Figs. 1, 2, 3.

**Table 1:** Patient and tumor characteristics

Variable	N_0_ (n = 45)	N_1–2_ (n = 16)	N_≥3_ (n = 20)	F/χ^2^	*P* values
Age (years)^a^	57.64 ± 10.04	58.44 ± 12.32	51.55 ± 10.31	2.706	0.073
Lesion size(cm)^b^	1.70 (0.70)	2.25 (1.38)	3.00 (1.15)	20.092	0.000
Tumor Position^c^				0.606	0.739
Outer upper	19 (42.2)	6 (37.5)	10 (50.0)		
Others	26 (57.8)	10 (62.5)	10 (50.0)		
ER^c^				0.462	0.479
Positive	34 (75.6)	15 (93.8)	16 (80.0)		
Negative	11 (24.4)	1 (6.2)	4 (20.0)		
PR^c^				0.649	0.723
Positive	29 (64.4)	12 (75.0)	13 (65.0)		
Negative	16 (35.6)	4 (25.0)	7 (35.0)		
HER2^c^				3.4145	0.065
Positive	6 (13.3)	1 (6.2)	7 (35.0)		
Negative	39 (86.7)	15 (93.8)	13 (65.0)		
Ki-67^c^				0.997	0.318
Positive	37 (82.2)	15 (93.8)	18 (90.0)		
Negative	8 (17.8)	1 (6.2)	2 (10.0)		
Molecular subtype^c^				9.800	0.133
Luminal A	8 (17.8)	1 (6.2)	2 (10.0)		
Luminal B	26 (57.8)	14 (87.5)	14 (70.0)		
HER2 positive	1 (2.2)	1 (6.2)	1 (5.0)		
Triple negative	10 (22.2)	0 (0)	3 (15.0)		
Histologic grade^c^				5.161	0.076
I/II	24 (53.3)	9 (56.2)	5 (25.0)		
III	21 (46.7)	7 (43.8)	15 (75.0)		

^a^Data are presented as mean value ± standard deviation

^b^Data are presented as median (interquartile range)

^c^Categorical variables are numbers with percentages in parentheses

*ER*  estrogen receptor, *PR*  progesterone receptor, *HER2*  human epidermal growth factor receptor-2

### Interobserver agreement assessment

The interobserver agreement for analyzing the histogram parameters produced from FOCUS DWI was excellent between the two radiologists (ICC values ranged from 0.919 to 0.982). The details are displayed in Table 2.

**Table 2:** Interobserver variability of ADC histogram parameters of FOCUS diffusion weighted imaging

Parameters	ICC	95% CI
Skewness	0.919	0.877–0.947
Median	0.972	0.957–0.982
Energy	0.982	0.972–0.988
Maximum	0.935	0.900–0.957
90 Percentile	0.925	0.886–0.951
Minimum	0.959	0.937–0.974
Range	0.928	0.890–0.953
10 Percentile	0.974	0.960–0.983
Kurtosis	0.936	0.902–0.958
Mean	0.973	0.958–0.983

*ICC* intra-class correlation coefficient, *CI* confidence interval

### Comparison of histogram parameters

The histogram parameters of energy, maximum, 90 percentile, and range revealed significant differences in breast cancers with different ALN involvement statuses (*P* = 0.000, 0.004, 0.017, and 0.001, respectively) (Table 3). The energy levels were significantly different between N_0_ and N_1–2_, whereas the energy, maximum, 90 percentile, range, and lesion size differed significantly between N_0_ and N_≥3_, and energy, maximum, 90 percentile, range, and lesion size differed significantly between N_1–2_ and N_≥3_ (all *P* < 0.05) (Table 4).

**Table 3:** ADC histogram parameters for differentiating ALN status

parameters	N_0_ (n = 45)	N_1–2_ (n = 16)	N_≥3_ (n = 20)	F/χ^2^	*P*
Skewness^b^	0.086 ± 0.802	0.192 ± 0.739	0.293 ± 0.610	0.549	0.580
Median^b^	0.669 ± 0.120	0.656 ± 0.130	0.710 ± 0.119	1.058	0.352
Energy^a^(× 10^–3^)	0.028 (0.026)	0.036 (0.068)	0.131 (0.085)	26.252	0.000
Maximum^b^	0.952 ± 0.192	0.970 ± 0.235	1.143 ± 0.222	6.039	0.004
90 Percentile^b^	0.829 ± 0.131	0.825 ± 0.187	0.950 ± 0.200	4.318	0.017
Minimum^b^	0.379 ± 0.183	0.370 ± 0.133	0.321 ± 0.170	0.811	0.448
Range^b^	0.573 ± 0.228	0.601 ± 0.258	0.823 ± 0.287	7.158	0.001
10 Percentile^b^	0.514 ± 0.146	0.527 ± 0.110	0.533 ± 0.084	0.177	0.838
Kurtosis^a^	2.981(1.352)	3.247(1.132)	3.059(1.469)	0.227	0.892
Mean^b^	0.669 ± 0.120	0.667 ± 0.132	0.724 ± 0.121	1.547	0.219

The ADC values are given the units of 10^−3^ mm^2^/s

^a^Data are presented as medians (interquartiles range)

^b^Data are presented as means standard deviations

**Table 4:** Pairwise comparison of ADC histogram parameters among the different ALN status

parameters	*P* value N_0_ vs N_1–2_	*P* value N_0_ vs N_≥3_	*P* value N_1–2_ vs N_≥3_
Skewness	0.629	0.307	0.688
Median	0.713	0.217	0.192
Energy	**0.039**	**0.000**	**0.013**
Maximum	0.760	**0.001**	**0.016**
90 Percentile	0.921	**0.007**	**0.023**
Minimum	0.859	0.212	0.395
Range	0.705	**0.000**	**0.010**
10 Percentile	0.731	0.576	0.880
Kurtosis	0.658	0.755	0.949
Mean	0.962	0.099	0.171
Lesion size	0.065	**0.000**	**0.014**

Significant differences were in bold

### Results of ROC curves

**Fig. 4:**
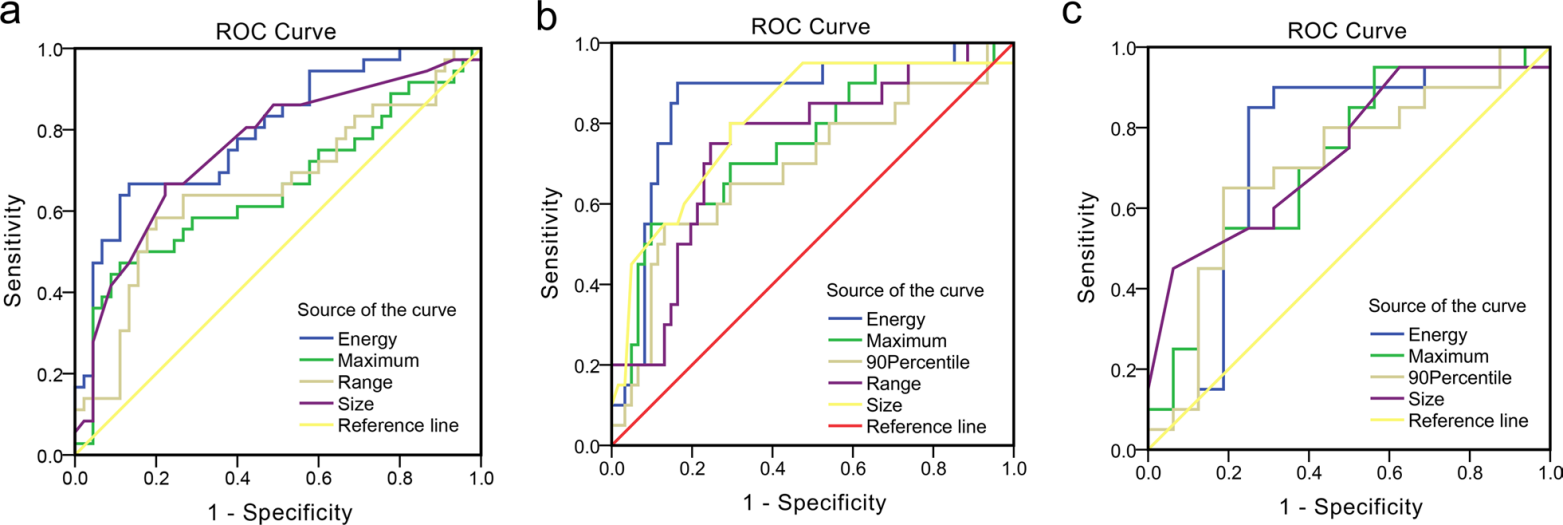
Receiver operating characteristic (ROC) curves of significant histogram parameters in differentiating ALN metastasis. **a** ROC curve of energy, maximum, range and lesion size for differentiation N_0_ versus N_1–2_ and N_≥3_. **b** ROC curve of energy, maximum, 90 Percentile, range and lesion size for differentiation N_0_ and N_1–2_ versus N_≥3_. **c** ROC curve of energy, maximum, 90 Percentile and lesion size for differentiation N_1–2_ versus N_≥3_

Figure 4 and Table 5 illustrate ROC curve analysis of relevant parameters. To predict ALN status between N_0_ and N_+_ (≥ 1 ALNs), AUC values of energy, maximum, range, and lesion size were 0.796, 0.654, 0.658, and 0.754, respectively, and the energy had the highest area under ROC curve. To differentiate N_0_ and N_1–2_ versus N_≥ 3_ groups, AUC values of energy, maximum, 90 percentile, range, and lesion size were 0.853, 0.746, 0.689, 0.745, and 0.809, respectively, and the energy had the highest area under ROC curve. To differentiate ALN status between N_1–2_ and N_≥ 3_ groups, AUC values of energy, maximum, 90 percentile, and lesion size were 0.744, 0.716, 0.703, and 0.739, respectively, and the energy had the highest area under ROC curve.

**Table 5:** The performance of significant histogram parameters for differentiating different ALN status

Comparison and parameter	AUC (95% CI)	*P* value
*N*_*0*_* versus N*_*1–2*_* and N*_≥*3*_		
Energy	0.796 (0.698–0.894)	0.000
Maximum	0.654 (0.529–0.780)	0.017
Range	0.658 (0.534–0.782)	0.015
Size	0.754 (0.645–0.863)	0.000
*N*_*0*_* and N*_*1–2*_* versus N*_≥*3*_		
Energy	0.853 (0.751–0.956)	0.000
Maximum	0.746 (0.616–0.876)	0.001
90 Percentile	0.689 (0.543–0.834)	0.012
Range	0.745 (0.619–0.871)	0.001
Size	0.809 (0.694–0.924)	0.000
*N*_*1–2*_* versus N*_≥*3*_		
Energy	0.744 (0.561–0.926)	0.013
Maximum	0.716 (0.544–0.888)	0.028
90 Percentile	0.703 (0.524–0.882)	0.039
Size	0.739 (0.577–0.901)	0.015

*AUC* area under the ROC curve, *CI* confidence interval

*p* values were calculated by using the Mann–Whitney U test

## Discussion

The current study demonstrated that the examination of ADC histogram for the whole lesion based on FOCUS DWI could be assessed as a non-invasive tool for evaluating the extent of ALN involvement in early-stage breast cancer patients. The findings disclosed that four histogram parameters and the lesion size differed significantly among early-stage breast cancers at different ALN statuses. Furthermore, according to ROC curve analysis, the energy value had the best diagnostic performance in differentiating N_0_ and N_1–2_ groups from N_≥ 3_ group with an AUC value of 0.853.

At present, the spin-echo single excitation (SS-EPI) sequence is commonly utilized for breast DWI acquisition. However, SS-EPI DWI images quality was not always satisfactory due to magnetic susceptibility artifacts [22]. In breast imaging, image distortions and artifacts are evident due to anatomical complexity and isocentric scans [23]. Our study obtained ADC maps for histogram analysis from FOCUS DWI sequences. FOCUS DWI is a new diffusion technique that uses two-dimensional space selective excitation pulses and 180˚ refocusing pulses [18]. A previous study reported that in breast cancer, FOCUS DWI prominently improves image quality with a reduction of artifacts [24]. In our study, the inter-observer variability study revealed that all parameters in the whole tumor histogram analysis had high inter-observer repeatability, with all parameters achieving ICCs greater than 0.900. However, a previous study [25] showed that skewness and kurtosis ICC scores were reasonably low (0.756 and 0.734, respectively) by demonstrating the efficacy of the whole-lesion technique based on SS-EPI DWI for discriminating Ki-67 expression in invasive breast cancer at T1 stage. To some extent, this phenomenon can be explained using FOCUS DWI technology; this enhances signal-to-noise ratio and spatial resolution of the picture, help overcome the partial volume effect, and thus make the whole tumor boundary delineation and semi-automatic segmentation results more accurate. Excellent inter-observer variability of whole-lesion histogram parameters depending on high-resolution FOCUS DWI technology is critical to ensuring the reliability of breast cancer quantification studies.

Breast cancers with different numbers of metastatic ALN require different surgical axillary treatment: patients with negative ALN metastasis do not require SLN biopsy or ALND. SLND is only for patients with 1–2 metastatic ALNs, and ALND is dedicated to patients with ≥ 3 metastatic ALNs [3, 26]. In our study, energy, maximum, 90 percentile, range, and lesion size revealed significant differences in predicting ALN status of early-stage breast cancer. Energy reflects the size of voxel value in the image. Prior research [27] showed that energy was significantly associated with histological grade and lymphovascular invasion of breast cancer. Zhao et al*.* proved that energy and total energy performed well in differentiating pN0 from pN1–2 nodal staging of the rectal cancer [28]. In our study, the energy showed better diagnostic efficacy than the other parameters, which to some extent indicates that the energy value might be more linked to the malignant degree and invasiveness of cancers. A higher value of range reflects more variation of the intensity within VOI. In our study, the range values in N_≥3_ groups were significantly greater than those in N_1–2_ and N_0_ groups. Therefore, the range can reflect tumor heterogeneity to a certain extent.

According to a previous study, lower percentiles represent dense tumor cells, while higher percentiles reflect the areas of necrotic and edema components [14]. In our study, the higher ADC percentiles (maximum, 90 percentile), corresponding to more necrotic and cystic components, showed closer correlations with ALN metastasis than lower ADC percentiles. Wang et al*.* [29] also identified that ADC 90 percentiles showed higher diagnostic efficacy for differentiating lymph node-positive and lymph node-negative groups of epithelial ovarian cancer. However, Liu et al*.* stated that lower ADC percentiles (such as 10 percentiles) showed more significant differences in gastric cancer patients with different N stages than higher percentiles [30]. The above phenomena demonstrate that the probability of metastatic lymph nodes might be closely linked to different components of the primary tumor [31]. The low ADC percentiles and high ADC percentiles played different roles in evaluating the prognosis of tumors in different parts. For breast cancer, tumors with more necrotic and cystic areas are more likely to have the aggressive biological behavior of ALN metastases.

Previous studies reported that tumor size was an independent prognostic factor of SLN [32]. Tumor size was proportional to axillary lymph node metastasis, and each 0.1 cm higher in tumor size resulted in 4.29 times more likely to have SLN metastasis in breast cancer [33]. Our study showed that the lesion size was the largest for the number of metastatic ALNs of > 3, followed by the number of metastatic ALNs of 1–2, and then by no metastasis, similar to previous studies. Therefore, rapid tumor growth accompanied higher malignancy, leading to ALN metastasis [34].

Numerous limitations are present in this investigation. First, the study is a retrospective analysis of data acquired from a prospective study, and there is inevitable patient selection bias. Second, the sample size for this study was rather small, and a greater sample size and multicenter data will be considered for ALN state evaluation in the future. Finally, we only used the traditional simple exponential model, which may lead to the bias of ADC values. In the future, we will attempt to add intravoxel incoherent motion, diffusion kurtosis imaging, and their obtained factors into our research.

## Conclusions

In conclusion, our investigation demonstrated that ADC histogram parameters for the whole lesion based on a high-resolution FOCUS DWI image could improve the diagnostic performance in forecasting the level of breast cancer involvement in its early stages, which may contribute to the selection of an appropriate therapeutic approach.

## Data Availability

The datasets used and/or analysed during the current study available from the corresponding author on reasonable request.
